# Bovine Dermal Collagen Matrix for Early Graft Readiness in a Full-Thickness Posterior Neck Burn: A Case Report

**DOI:** 10.7759/cureus.98818

**Published:** 2025-12-09

**Authors:** Cameron Gibson, Amber Kohler, Thomas Vogler, Arek Wiktor

**Affiliations:** 1 Department of Surgery, University of Colorado Anschutz School of Medicine, Aurora, USA

**Keywords:** bovine dermal collagen matrix (bdcm), burn injury, collagen scaffold, dematology, dermal substitute, early graft readiness, full-thickness burn reconstruction, matrix integration, rapid vascularization, split-thickness skin graft (stsg) wound bed preparation

## Abstract

Full-thickness burns over complex anatomical regions present significant challenges for achieving wound closure. In these cases, dermal substitutes are critical to support a dermal-like bed formation to optimize graft outcomes. Here, we present the case of a 63-year-old male patient with a full-thickness posterior neck burn treated with a bovine dermal collagen matrix (BDCM) engineered to support rapid vascularization. Following surgical excision and wound bed preparation, BDCM was applied with minimal stapling and covered with standard antimicrobial dressings. In this challenging wound, early matrix integration was evident by postoperative day 6, and the wound demonstrated graft readiness by approximately day 12. Definitive closure was achieved with split-thickness skin grafting on day 16, with complete epithelialization observed at day 11 post-grafting. The patient experienced full graft take, intact wound healing, and restoration of neck mobility without infection or early contracture, indicating a successful clinical outcome. This initial experience demonstrates rapid wound bed preparation with BDCM, stable graft take, and successful healing with minimal complications, showcasing BDCM’s potential as a valuable dermal substitute for facilitating definitive closure in challenging full-thickness wounds.

## Introduction

In full-thickness burn injuries, particularly those involving large surface areas, mobile regions, or anatomically complex sites, dermal matrices are often utilized to promote the formation of a stable, vascularized dermal-like wound base. This intermediate layer reduces wound contraction, enhances graft take, and supports more reliable and durable wound closure [1]. Traditional methods of direct grafting onto poorly vascularized beds can result in graft loss or hypertrophic scarring [2,3].

Cohealyx^TM^ (AVITA Medical, Inc., Santa Clarita, California, United States) is a bovine dermal collagen matrix (BDCM) derived from young bovine dermis and composed of Type I and Type III collagen. The BDCM is crosslinked to optimize scaffold strength and control matrix degradation in the wound bed. The matrix functions as a scaffold that promotes cellular infiltration and revascularization [4,5].

Preclinical models have demonstrated the ability of BDCM to promote rapid vascularization, superior integration, and early readiness for definitive closure compared to other collagen-based dermal matrices. This case is presented to demonstrate the reconstructive decision-making involved in managing a full-thickness posterior neck burn, a region challenged by constant motion and high contracture risk. It illustrates how a staged approach using a dermal collagen matrix supported wound bed preparation and timely autografting. Here, we report the first clinical use of BDCM at our institution in a patient with a full-thickness wound on the posterior neck following excision of a thermal injury.

## Case presentation

**Figure 1:**
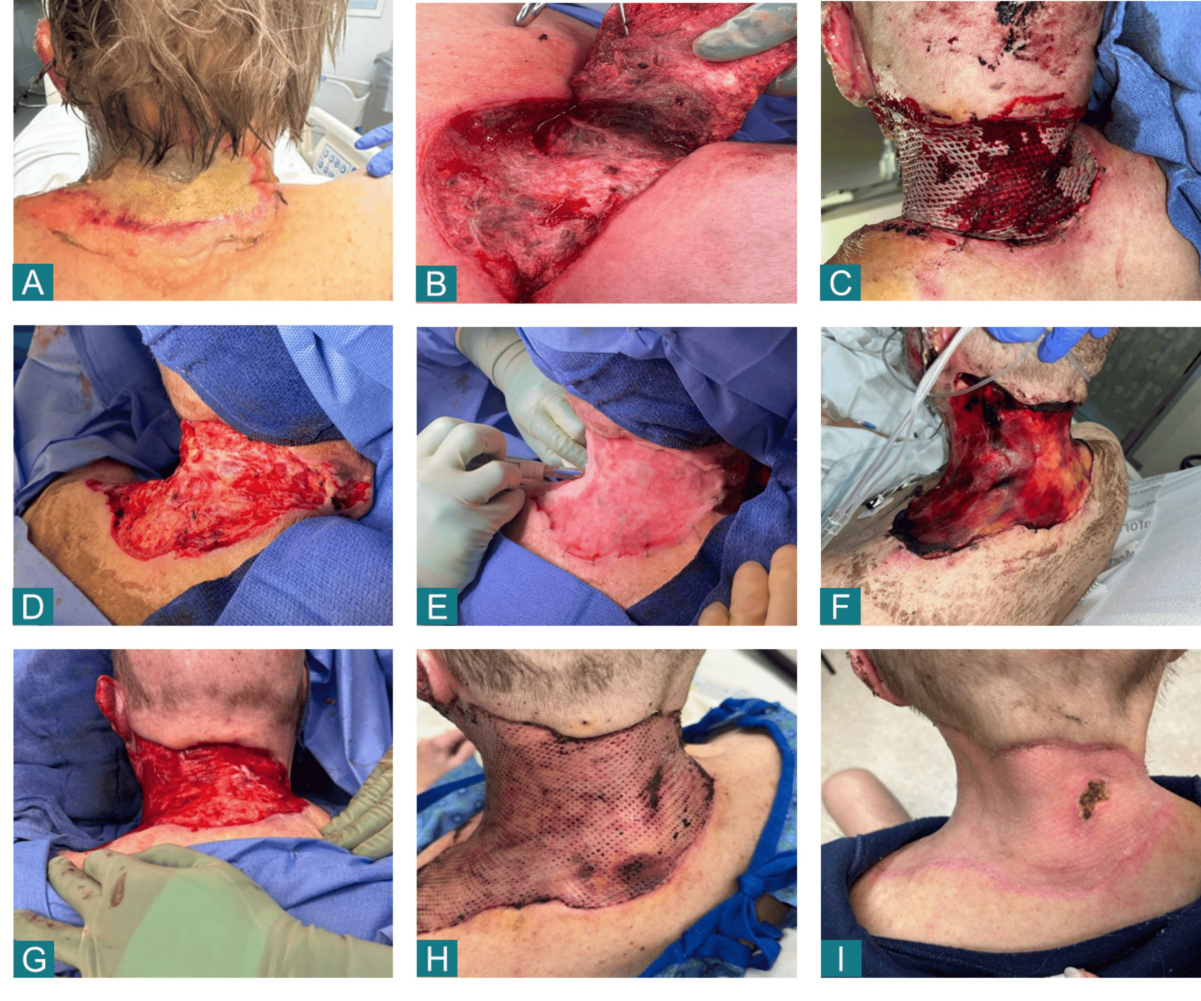
Sequential Wound Healing Following BDCM Application in a Full-Thickness Posterior Neck Burn. (A) Initial presentation demonstrating thick eschar over the posterior cervical region. (B) Intraoperative view of exposed wound bed demonstrating the depth of nonviable tissue and peripheral viability. (C) Temporary meshed allograft placement after initial excision.  (D) Final wound bed preparation showing a healthy, vascularized surface. (E) Application of hydrated Cohealyx matrix with slight overlap and minimal stapling. (F) Postoperative day 6 after Cohealyx application, demonstrating a matte appearance and early color variation. (G) Wound bed at time of grafting, showing robust granulation after Cohealyx-supported healing. (H) Early postoperative appearance following split-thickness skin graft (STSG) placement. (I) Final healing outcome with complete epithelialization and maturing scar approximately five weeks after initial matrix placement. BDCM: bovine dermal collagen matrix

A 63-year-old Caucasian man presented to the Burn and Frostbite Center with a third-degree contact burn involving the posterior neck and extending to the right shoulder, affecting approximately 400 cm² of surface area (Figure 1A). His medical history included alcohol use disorder, hypertension, and coronary artery disease. The burn was of unknown etiology, and on admission, the patient exhibited thick leathery eschar across the posterior neck. No signs of active infection were noted following initial topical soaks. Given the depth and location of the wound, surgical intervention was deemed necessary to excise nonviable tissue and initiate reconstruction.

Initial surgical management

The patient underwent tangential excision (Figure 1B) followed by placement of a temporary meshed allograft (Figure 1C). Partial failure of the temporary meshed allograft was observed over the following days, prompting a second debridement. After excising the remaining nonviable tissue, a clean full-thickness wound bed was established (Figure 1D).

BDCM application

Two sheets of BDCM (20 cm × 10 cm each) were applied to cover the posterior cervical defect (~400cm²) (Figure 1E). BDCM sheets were hydrated with sterile normal saline directly on the wound surface until pliable, then carefully molded to the wound contours. A slight overlap was created between sheets to ensure continuous coverage while minimizing excessive stacking. To ensure complete apposition, the matrix was manually contoured to eliminate air pockets. Minor intraoperative bleeding occurred during hydration and was controlled using targeted sutures without disrupting matrix placement. The matrix was fixed with perimeter staples spaced approximately 1 inch apart, ensuring stability while preserving matrix integrity. A multilayer dressing was then applied, consisting of a non-adherent transparent contact layer, a single layer of silver-impregnated antimicrobial dressing, followed by a soft gauze roll, an absorbent burn pad, and an outer compression dressing. Dressing takedown was planned at postoperative day (POD) 6, with interim monitoring for signs of bleeding, dryness, or matrix detachment.

Matrix integration and wound progression

By POD 6, BDCM displayed a matte appearance with color variations (red, pink, peach), consistent with an early healthy wound bed (Figure 1F). In response to signs of mild wound dryness, the dressing protocol was modified to a thin layer of bacitracin ointment beneath adaptic gauze to maintain appropriate moisture levels.

Grafting and outcome

On POD 16, the BDCM-prepared wound bed was lightly debrided with Versajet (Smith & Nephew plc, Watford, United Kingdom) (Figure 1G), and a 1:1 split-thickness skin graft (STSG) was placed. The skin graft was then dressed with Telfa™ clear, bacitracin-aden xeroform gauze, burn wound pad (Cardinal Health, Inc., Dublin, Ohio, United States), and a Silverlon compression dressing (Bravida Medical, Geneva, Illinois, United States). Removal of the surgical dressing on POD 4 revealed excellent graft adherence without hematoma or infection. Full epithelialization was noted by POD 11 post-grafting (Figure 1H), and by approximately 37 days post BDCM application, the wound achieved full closure (Figure 1I).

## Discussion

This case highlights the successful application of BDCM in managing a complex posterior cervical full-thickness burn. Posterior neck burns are particularly challenging due to anatomical mobility, proximity to lymphatic drainage, and difficulty maintaining stable dressings. BDCM demonstrated excellent conformability, mechanical integrity, and clinical performance in a complex wound.

BDCM supported healthy granulation tissue formation, achieving graft readiness within approximately two weeks, faster than what is typically observed with traditional dermal matrices, where time to grafting often exceeds three weeks [6,7]. Minor matrix fragility post-hydration did not negatively impact overall integration. Furthermore, successful graft take and early epithelialization were achieved with minimal wound bed preparation at the time of grafting.

Although platelet-rich plasma and other autologous skin cell suspension concentrates have been reported in the literature as adjuncts for enhancing graft outcomes, these modalities were not used in this case [8,9]. The successful progression of the wound bed and the favorable postoperative outcome demonstrate that, for selected patients, a collagen-based matrix alone may provide sufficient support for achieving graft readiness in challenging anatomic regions.

This report contributes to the clinical understanding of staged reconstruction in posterior neck burns and highlights the potential utility of dermal matrices in preparing complex wounds for successful autografting. These clinical outcomes align with preclinical findings, which demonstrated that BDCM supports faster vascularization and earlier wound closure compared to other collagen-based matrices [5]. Importantly, the ability to expedite definitive closure has the potential to reduce morbidity, hospital length of stay, and healthcare costs.

## Conclusions

In this case, BDCM facilitated early wound bed preparation and successful grafting in a full-thickness posterior neck burn. The matrix provided strong biological support, enabling durable wound closure with minimal complications. These findings suggest that BDCM may offer clinical advantages in managing complex full-thickness wounds in a timely fashion. Further prospective studies are warranted to validate these findings across broader patient populations and in comparison to other dermal matrices available on the market.
